# Modulation of Biofilm Mechanics by DNA Structure and Cell Type

**DOI:** 10.1021/acsbiomaterials.2c00777

**Published:** 2022-10-27

**Authors:** Dawid Łysik, Piotr Deptuła, Sylwia Chmielewska, Karol Skłodowski, Katarzyna Pogoda, LiKang Chin, Dawei Song, Joanna Mystkowska, Paul A. Janmey, Robert Bucki

**Affiliations:** † Institute of Biomedical Engineering, Bialystok University of Technology, 15-351 Bialystok, Poland; ‡ Department of Medical Microbiology and Nanobiomedical Engineering, Medical University of Bialystok, 15-222 Bialystok, Poland; § Institute of Nuclear Physics, Polish Academy of Sciences, 31-342 Krakow, Poland; ∥ Department of Biomedical Engineering, Widener University, Chester, Pennsylvania 19087, United States; ⊥ Institute for Medicine and Engineering, University of Pennsylvania, Philadelphia, Pennsylvania 19104, United States

**Keywords:** deoxyribonucleic acid, rheology, compression-stiffening, mechanobiology

## Abstract

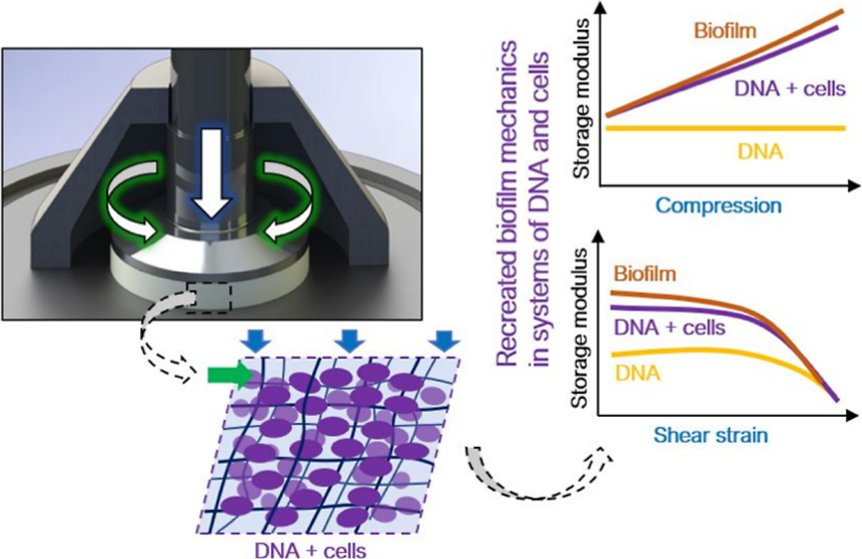

Deoxyribonucleic acid (DNA) evolved as a tool for storing and transmitting genetic information within cells, but outside the cell, DNA can also serve as “construction material” present in microbial biofilms or various body fluids, such as cystic fibrosis, sputum, and pus. In the present work, we investigate the mechanics of biofilms formed from *Pseudomonas aeruginosa* Xen 5, *Staphylococcus aureus* Xen 30, and *Candida albicans* 1408 using oscillatory shear rheometry at different levels of compression and recreate these mechanics in systems of entangled DNA and cells. The results show that the compression-stiffening and shear-softening effects observed in biofilms can be reproduced in DNA networks with the addition of an appropriate number of microbial cells. Additionally, we observe that these effects are cell-type dependent. We also identify other mechanisms that may significantly impact the viscoelastic behavior of biofilms, such as the compression-stiffening effect of DNA cross-linking by bivalent cations (Mg^2+^, Ca^2+^, and Cu^2+^) and the stiffness-increasing interactions of *P. aeruginosa* Xen 5 biofilm with Pf1 bacteriophage produced by *P. aeruginosa*. This work extends the knowledge of biofilm mechanobiology and demonstrates the possibility of modifying biopolymers toward obtaining the desired biophysical properties.

## Introduction

Deoxyribonucleic acid (DNA) is one of the most intensively studied organic compounds. In addition to storing and encoding genetic information, DNA is interesting from a materials science and engineering perspective.^1,2^ DNA molecules’ unique physical and chemical properties render them an essential building component for DNA-based generic materials.^3^ In many natural biological structures, DNA acts as a construction material. The best examples are biofilms, where microbial cells are embedded in a polymer matrix of extracellular polymeric substances (EPSs). EPS consists mainly of extracellular DNA (eDNA) and polysaccharides, which protect cells against mechanical forces, host immune systems, and antimicrobial agents, including exogenous antibiotics.^4^ While most research has focused on the mechanics of DNA,^5^ cells,^6^ or biofilms and its components,^7^ independently, the physics underlying the emergent rheological behavior of cell–DNA composites is still unclear. Research in this area is crucial to further our understanding of biofilm mechanobiology, the rheological properties of biopolymers, and to develop new strategies to control pathogens.

In recent years, mechanobiology has gained significant importance in medicine, and the measurement of mechanical properties has become the basis of numerous diagnostic methods, such as ultrasound,^8^ magnetic resonance elastography,^9^ and shear rheometry for histological assessment.^10^ Previous studies have established that substrate elasticity affects fundamental cellular processes, such as spreading, growth, cell division, differentiation, and migration of both eukaryotic and prokaryotic cells.^11−14^ Mechanical stresses can shape the behavior of not only tissue cells but also other biological systems such as biofilms. Just as compressional forces acting on the bone lead to tissue remodeling and increased mechanical properties, shear forces exerted on the biofilm lead to increased production of EPS, which enhances the integrity of the biofilm, likely as a survival strategy of the microbial culture.^15^ In this respect, biofilm is an archetype of tissues, containing cells that are embedded in and capable of remodeling the extracellular matrix.^16^ It is hypothesized that eDNA plays a major role in biofilm remodeling to environmental mechanical forces.^17,18^ In CF airways, the main source of eDNA in the biofilm matrix is neutrophils that release extracellular traps.^19^ eDNA is also released through bacterial cell autolysis via quorum sensing or altruistic suicide and during infection-induced necrosis of neutrophils and epithelial cells.^20^ An increased eDNA-to-cells ratio strengthens the structure of the biofilm and increases its viscoelastic moduli.^15^ In biological systems, where cells are embedded in an extracellular polymer matrix with complex stress states consisting of simultaneous compressive and shear forces, a critical threshold number of cells determines compression-stiffening.^21^ It remains unknown how the eDNA-to-cell ratio affects mechanics, and whether these mechanisms are dependent on the cell type. To address this question, we designed an experiment in which an entangled network of DNA and different types of microbial cells (*Pseudomonas aeruginosa* Xen 5, *Staphylococcus aureus* Xen 30, and *Candida albicans* 1408) with variable DNA-to-cell ratios was subjected to simultaneous compressive and shear forces. The results indicate that biofilm mechanics depends on the concentration of DNA and the morphology of the microorganisms.

Biofilms are complex and so are their mechanics. Cellular elements, as well as organic and inorganic components, interact with each other, resulting in the observed viscoelasticity (a combination of a viscous liquid-like response, quantified here by the shear loss modulus *G*″, and the solid-like elastic response quantified by the shear storage modulus *G*′) or compression-stiffening (increase in elastic storage modulus under compression). Based on studies of the compression-stiffening phenomenon in biological materials^22^ and factors influencing biofilm integrity,^23,24^ we expanded our investigation of the biofilm mechanics to include DNA cross-linking by bivalent cations and the interaction of biofilm components with filamentous bacteriophages secreted by *P. aeruginosa* Xen 5.

## Materials and Methods

### Bacterial and Fungal Cells

Three reference isolates, including the bacteria *S. aureus* Xen 30 and *P. aeruginosa* Xen 5 (Caliper Life Sciences, Hopkinton, MA, USA), and the fungus *C. albicans* 1408 (Polish Collection of Microorganisms, Polish Academy of Science, Wroclaw, Poland) were used. The analyzed strains were cultured and maintained on the recommended selective or selective-differential media, that is, Chapman agar (Biomaxima, Poland) for *S. aureus* Xen 30, Cetrimide agar (Thermo Scientific Oxoid, USA) for *P. aeruginosa* Xen 5, and Sabouraud Dextrose agar with chloramphenicol (Biomaxima, Poland) for *C. albicans* 1408. Bacterial cells of *S. aureus* Xen 30 and *P. aeruginosa* Xen 5 were cultured in an LB broth (Biomaxima, Poland), whereas *C. albicans* 1408 was grown in a RPMI medium supplemented with MOPS and d-(+)-glucose (all from Sigma-Aldrich, USA) to mid-log phase at 37 °C in aerobic conditions to a final cell density of 10^8^ colony-forming unit (cfu)/mL. At the end of incubation, an inoculum of microorganisms was centrifuged at 2000 rpm for 10 min. The supernatant was discarded and the sedimented bacteria or fungi were used for further investigation.

### Biofilm Formation

The biofilm was grown in 92 × 16 mm Petri dishes using a method that is widely used in the literature.^25^ Single colonies of *S. aureus* Xen 30, *P. aeruginosa* Xen 5, and *C. albicans* 1408 from an overnight culture of 18–24 h at 37 °C on Chapman agar, cetrimide agar, and Sabouraud Dextrose agar with chloramphenicol, respectively, were suspended in sterile broths to a cell density corresponding to 10^8^ cfu/mL. The biofilm created by *S. aureus* Xen 30 and *P. aeruginosa* Xen 5 was grown in LB, while the biofilm formed by *C. albicans* 1408 was grown in a RPMI medium supplemented with MOPS and d-(+)-glucose. After 72 h incubation at 37 °C in aerobic conditions without shaking, each Petri dish was washed with phosphate-buffered saline (PBS, Sigma-Aldrich, USA) to remove planktonic cells, and the mature biofilm was carefully collected from the Petri dish with a spatula. Excess buffer was removed using membrane filters.

### DNA Solutions with Microbial Cells

In the first step, DNA sodium salt from salmon testes (Sigma-Aldrich, USA) was dissolved at a concentration of 25 mg/mL in PBS. To obtain the desired concentration of cells in DNA, DNA, cells, and PBS were mixed at appropriate ratios (Table 1) and homogenized. The final DNA concentration was kept constant at 12.5 mg/mL. For comparison, the average concentration of DNA in CF sputum was reported in the range is 0.2–20 mg/mL.^26−30^

**Table 1:** Ratio of Components Used (in Units of Volume) to Obtain a Mixture of DNA and Cells

sample	DNA	cells	PBS
DNA + 0% cells	0.5	0	0.5
DNA + 10% cells	0.5	0.1	0.4
DNA + 30% cells	0.5	0.3	0.2
DNA + 50% cells	0.5	0.5	0

### DNA Solutions with Cations

DNA solutions with bivalent cations and cells were prepared similarly to DNA solutions with cells but without bivalent cations. DNA sodium salt from salmon testes (Sigma-Aldrich, USA) was dissolved to a concentration of 25 mg/mL in PBS and then aqueous solutions of magnesium, calcium, and copper chloride salts (Chempur, Poland) were added to obtain a 10 mM concentration (for comparison the concentration in the cystic fibrosis sputum is about 1.23 mM for magnesium, about 2.5 mM for calcium and 2.72 μM for copper ions^31^). The final DNA concentration was 12.5 mg/mL.

### Biofilm with Pf1 Bacteriophages

A mature biofilm of *P. aeruginosa* Xen 5 incubated for 72 h was homogenized with solutions of Pf1 bacteriophage (ASLA Biotech, Latvia) in PBS to obtain various concentrations of Pf1 in the biofilm (0.5, 1.0, and 2 mg/mL).

### Rheological Characteristics

**Figure 1:**
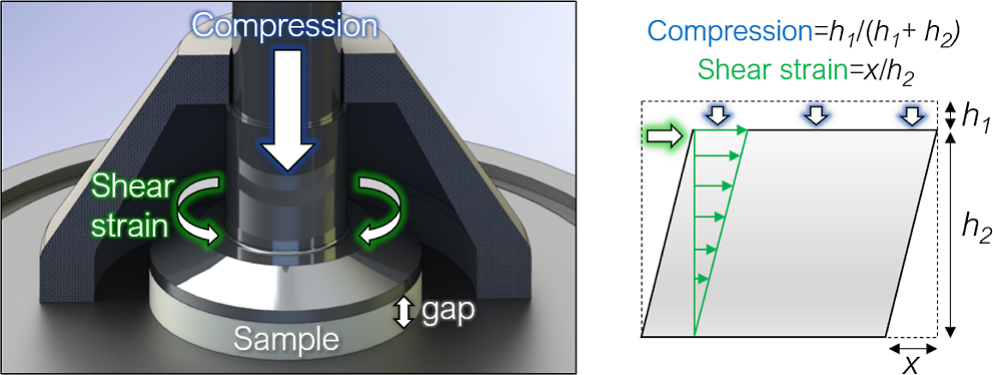
Parallel-plate strain-controlled, rotational shear rheometer was used to apply oscillatory shear strain by rotation of the upper platen. Compression was applied by a step-wise decrease of gap height between plates. Shear stress (the ratio of the shear force induced by the rotation of the upper plate to the cross-sectional area of the sample) and axial stress (here, the ratio of the compressive force exerted on the sample to the cross-sectional area of the sample) were measured. A solvent trap was used to prevent excessive evaporation (for better visibility, only half of the trap is shown in the figure).

The rheological properties of DNA solutions were measured on a parallel-plate, strain-controlled, rotational shear rheometer (HAAKE Rheostress 6000, Thermo Fisher Scientific, USA) (Figure 1). The volume of each sample for rheological studies was 300 μL and the upper platen diameter was 20 mm. DNA or biofilm samples were gently applied with a micropipette to the bottom plate of the rheometer and shielded from evaporation by a solvent trap. After setting the initial gap, the sample was left to relax for 2 min and reach a uniform temperature. The rheological testing protocol consisted of the following: 1) oscillating shear strain with a frequency *f* = 1 Hz and an amplitude γ = 1% for 60 s at each compression level (ε = 0, 10, 20, 30, 40, 50%) applied by the step-wise decrease in the gap height between the plates and 2) shear strain amplitude sweeps of γ = 0.1–100% with a frequency *f* = 1 Hz of the uncompressed (ε = 0%) and compressed (ε = 50%) samples. The storage modulus as a function of frequency is shown in Figure S1. The results are the mean of the measurements of three samples. The slope of the axial stress at various compression levels was used to calculate the apparent Young’s modulus (*E*) (defined as the ratio of axial stress to compression) (Table 2).

**Table 2:** Apparent Young’s Modulus and Slope of *G*′ Storage Modulus for Cells and DNA Composition during Compression^a^

	E (Pa)	adj. *R*^2^	*G*′ slope (Pa/%)	adj. *R*^2^
DNA + 30% PA cells	108 ± 2	0.99	0.59 ± 0.05	0.97
DNA + 50% PA cells	220 ± 8	0.99	1.30 ± 0.11	0.96
DNA + 30% SA cells	30 ± 1	0.99	0.22 ± 0.01	0.99
DNA + 50% SA cells	86 ± 2	0.99	0.61 ± 0.04	0.98
DNA + 30% CA cells	64 ± 1	0.99	0.41 ± 0.04	0.96
DNA + 50% CA cells	131 ± 2	0.99	0.98 ± 0.09	0.96

a Mean values ± standard error.

### Statistical Analysis

Statistical analyses were performed using OriginPro 2020 (OriginLab Corporation, Northampton, USA). All presented data are mean ± SD. The significance of differences was determined using the one-way ANOVA. *p* < 0.05 was considered to be statistically significant.

## Results

**Figure 2:**
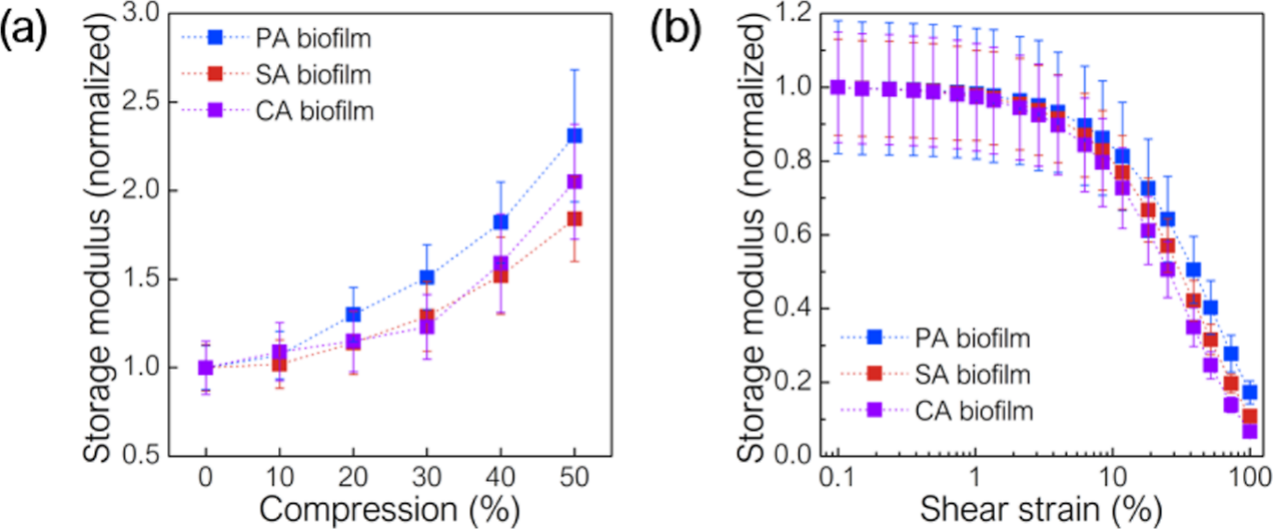
Normalized storage modulus of PA, SA, and CA biofilms as a function of (a) compression and (b) shear strain. Biofilm exhibits compression-stiffening (increase in the storage modulus during compression) and shear strain-softening (decrease in the storage modulus with increasing shear strain amplitude). The absolute values of the storage modulus are shown in Figure S2.

Herein, we assess the rheological behavior of mature biofilms of *P. aeruginosa* Xen 5 (PA), *S. aureus* Xen 30 (SA), and *C. albicans* 1408 (CA) using oscillatory tests with 1% shear deformation at 1 Hz at various compression levels (ε = 0–50% in 10% steps) and shear strain sweeps (0.1—100%, 6 steps per decade) at 1 Hz. The stiffness of biofilms, as measured by the shear storage modulus (the ratio of the elastic shear stress to shear strain, indicating the material’s ability to store strain energy), non-linearly increases by 1.8–2.3 times at 50% axial deformation (Figure 2a). Biofilm stiffness decreases with a shear strain of 10% or greater (Figure 2b). At 100% shear strain, stiffness decreases to 5–20% of its initial value. Such rheological behavior can be interpreted as the biofilm increasing resistance to mechanical stress acting normal to its surface but having the ability to move with shear.

**Figure 3:**
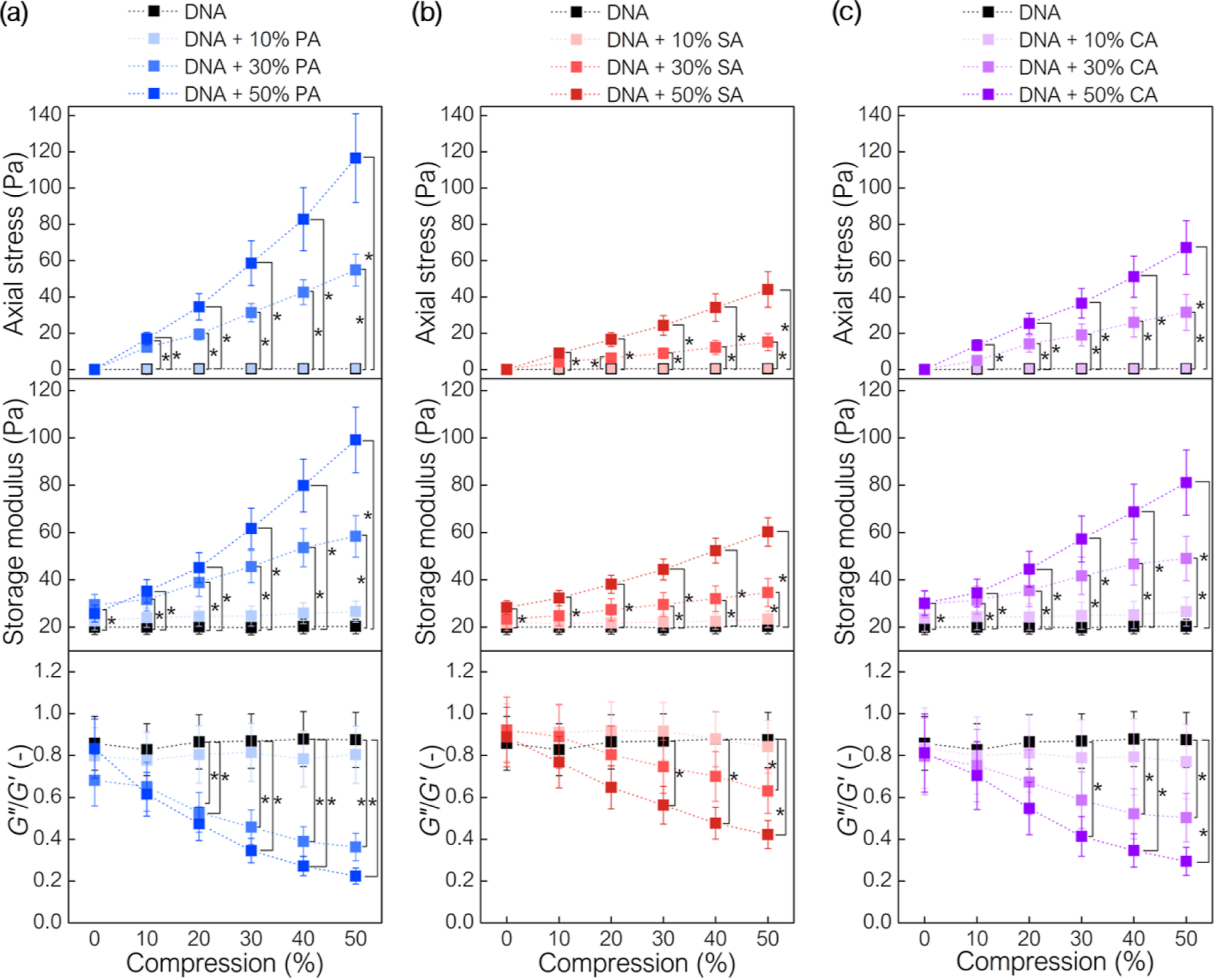
Rheological properties of the DNA–cell: (a) *P. aeruginosa* Xen 5, (b) *S. aureus* Xen 30, and (c) *C. albicans* 1408 networks subjected to simultaneous compression and oscillating shear. The concentration and type of cells in the DNA network influenced the rheological behavior during compression. Compression-stiffening increased with the volume fraction of cells and was most evident for PA cells. (*) indicates statistical significance (*p* < 0.05).

The biofilm consists of cells and extracellular substances, including eDNA. We examined how biofilm mechanics can be recapitulated in a simple system containing cells and DNA. Based on previous works on tissue mechanics and modeling using a combination of nonlinear elastic polymer networks with cell inclusions,^32^ we performed a series of rheological experiments using different types of microbial cells suspended in a constant concentration of DNA (12.5 mg/mL)—small (measuring 0.5 to 0.8 by 1.5 to 3.0 μm, average area 1.24 μm^2^), rod-shaped (aspect ratio 1.96; roundness 0.52) PA, small (average area 0.99 μm^2^) and round (aspect ratio 1.19; roundness 0.95) SA, and large (measuring from 3 to 6 by 1 to 3 μm, average area 15.96 μm^2^) and oval (aspect ratio 1.13; roundness 0.89) CA cells (Figure S3). Figure 3 presents the results of simultaneous compression and oscillating shearing of the entangled network of DNA and cells of *P. aeruginosa* Xen 5 (Figure 3a), *S. aureus* Xen 30 (Figure 3b), and *C. albicans* 1408 (Figure 3c) where axial stress, storage modulus *G*′, and the ratio of loss modulus *G*″ (the ratio of the viscous shear stress to shear strain, indicating the material’s ability to dissipate strain energy) to *G*′ as a function of compression are shown. The axial stress and storage modulus relaxation over time is shown in Figure S4a,b.

Step-wise compression (0–50%) does not change the storage or loss moduli of DNA networks without cells. The storage modulus is approximately 20 Pa, and the *G*″/*G*′ ratio is about 0.86. Although the addition of 10% microbial cells to the DNA network did not significantly change the mechanics, the addition of 30% microbial cells did. For SA cells, axial stress and storage modulus significantly increase at compression levels 30% and above, reaching 15 and 35 Pa at 50% compression, respectively. The apparent Young’s modulus of this composition is 30 Pa. The *G*″/*G*′ ratio decreases linearly from ∼0.92 at 0% compression to ∼0.63 at 50% compression, showing an increase in the relative elasticity of the solutions with increasing compression. The effect of 30% cells on the rheology of PA- and CA-DNA samples was pronounced (significant difference in axial stress and storage modulus at 10% and above compression). For both cell types, axial stress increases linearly—to 55 Pa for PA cells and 32 Pa for CA cells, which allows for determining the apparent Young’s modulus at 108 and 64 Pa, respectively. Similarly, the storage modulus of DNA networks increases linearly with the addition of 30% cells—from 29 to 58 Pa for PA cells and from 30 to 49 Pa for CA cells. A decrease in the *G*″/*G*′ ratio is observed for both PA and CA cells, decreasing from 0.68 (0% compression) to 0.36 (50% compression) and from 0.80 (0% compression) to 0.50 (50% compression), respectively. With a cell volume fraction of 50%, the mechanics of the DNA network changes radically, showing a significant increase in both axial stress and storage modulus with compression. Compression-stiffening is pronounced for PA rod-shaped cells, where the apparent Young’s modulus is 220 Pa, and the storage modulus increases from 26 to 99 Pa. Marked compression-stiffening is also observed for 50% CA-DNA networks, where axial stress increases to 67 Pa and the storage modulus from 30 to 81 Pa. The apparent Young’s modulus of this composition is 131 Pa. The smallest increase in mechanical properties was for SA cells—axial stress increases to 44 Pa at 50% compression (*E* = 86 Pa), while the storage modulus increases from 28 at 0% compression to 60 Pa at 50% compression. The *G*″/*G*′ ratio decreases from 0.83 at 0% compression to 0.22 at 50% compression for PA, from 0.89 to 0.42 for SA, and from 0.81 to 0.29 for CA.

**Figure 4:**
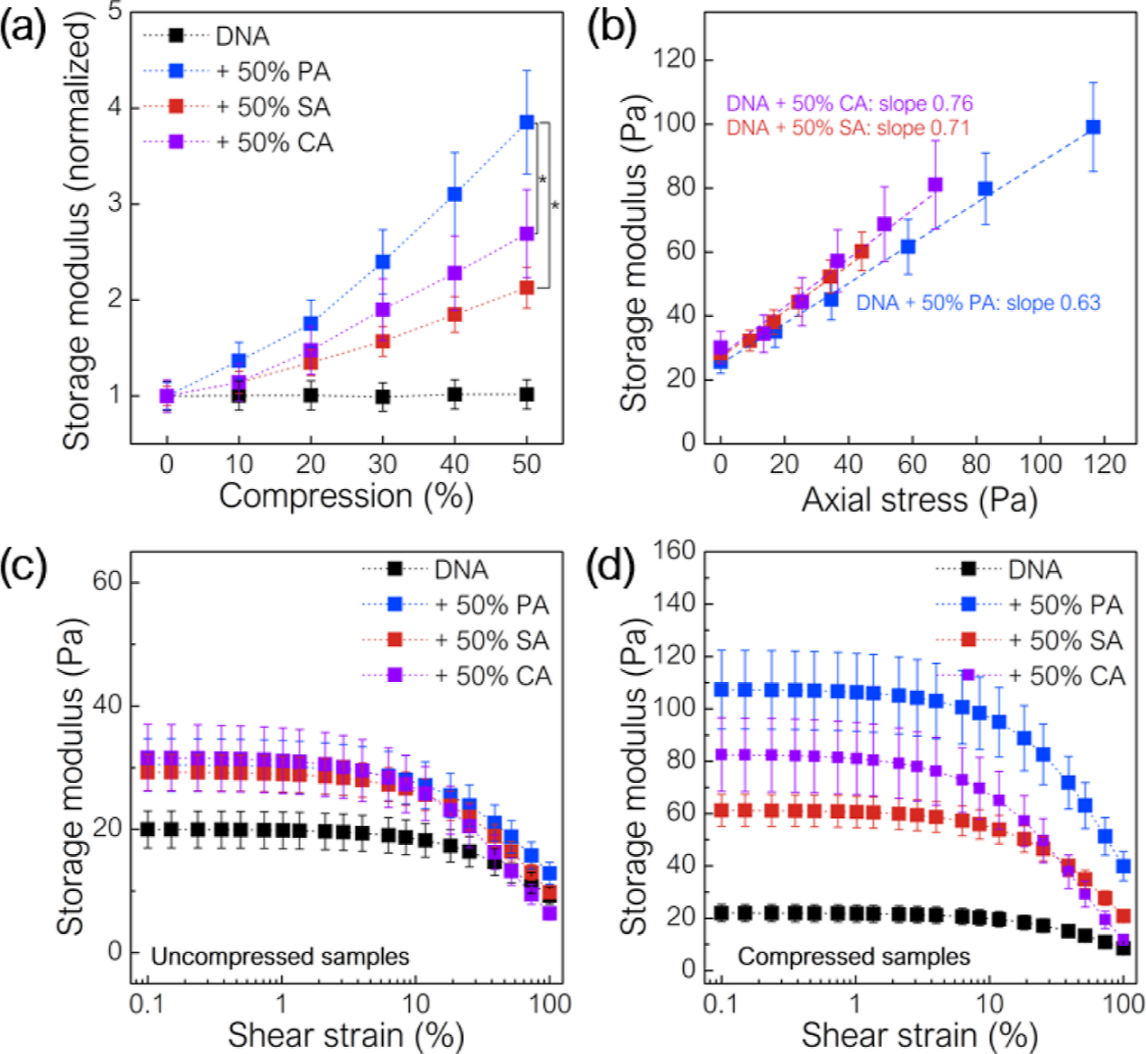
Rheological properties of DNA networks with and without cells subjected to simultaneous compression and oscillatory shear: (a) normalized storage modulus for DNA systems with and without 50% cellular content, (b) storage modulus as a function of axial stress, (c) storage modulus as a function of shear strain amplitude for uncompressed DNA samples with and without 50% cellular content, and (d) storage modulus as a function of shear strain amplitude for compressed DNA samples with and without 50% cellular content (replotted data). (*) indicates statistical significance (*p* < 0.05).

Our results indicate that the presence of microbial cells in the DNA network changes its mechanical response during compression, and compression-stiffening increases with the cell number and depends on the cell type. The greatest apparent Young’s modulus and relative increase in the storage modulus were observed for the DNA network with PA cells (Table 2) (Figure 4a). An increase in the storage modulus for the PA–DNA network was also greater than that observed in the PA biofilm (*p* < 0.02). For DNA with 50% of cells, the storage modulus as a function of axial stress is a straight line with a slope of 0.62 for PA, 0.72 for SA, and 0.76 for CA (Figure 4b).

Storage modulus as a function of shear strain is plotted for uncompressed and compressed (50% axial deformation) DNA networks with and without 50% cellular content (Figure 4c,d). Both uncompressed and compressed samples exhibit shear-softening, with initial values of storage modulus that correspond to the moduli obtained during compression tests with constant strain amplitude (Figure 3). In comparison to the biofilm, the transition point for DNA–cell systems occurs at higher shear strains, perhaps due to the mechanical contribution of DNA, which itself exhibits a higher breaking point. Additionally, the extent of shear-softening is much greater for biofilms (>80% average decrease) than for DNA–cell networks (Table 3).

**Table 3:** Shear-Softening Effect of Uncompressed and Compressed DNA and Cell Compositions

	uncompressed		compressed	
	*G*′ initial value (Pa)	average decrease at 100% shear strain (%)	*G*′ initial value (Pa)	average decrease at 100% shear strain (%)
DNA	20	54	33	61
DNA + 50% PA	30	58	107	63
DNA + 50% SA	30	66	61	66
DNA + 50% CA	32	80	83	86

**Figure 5:**
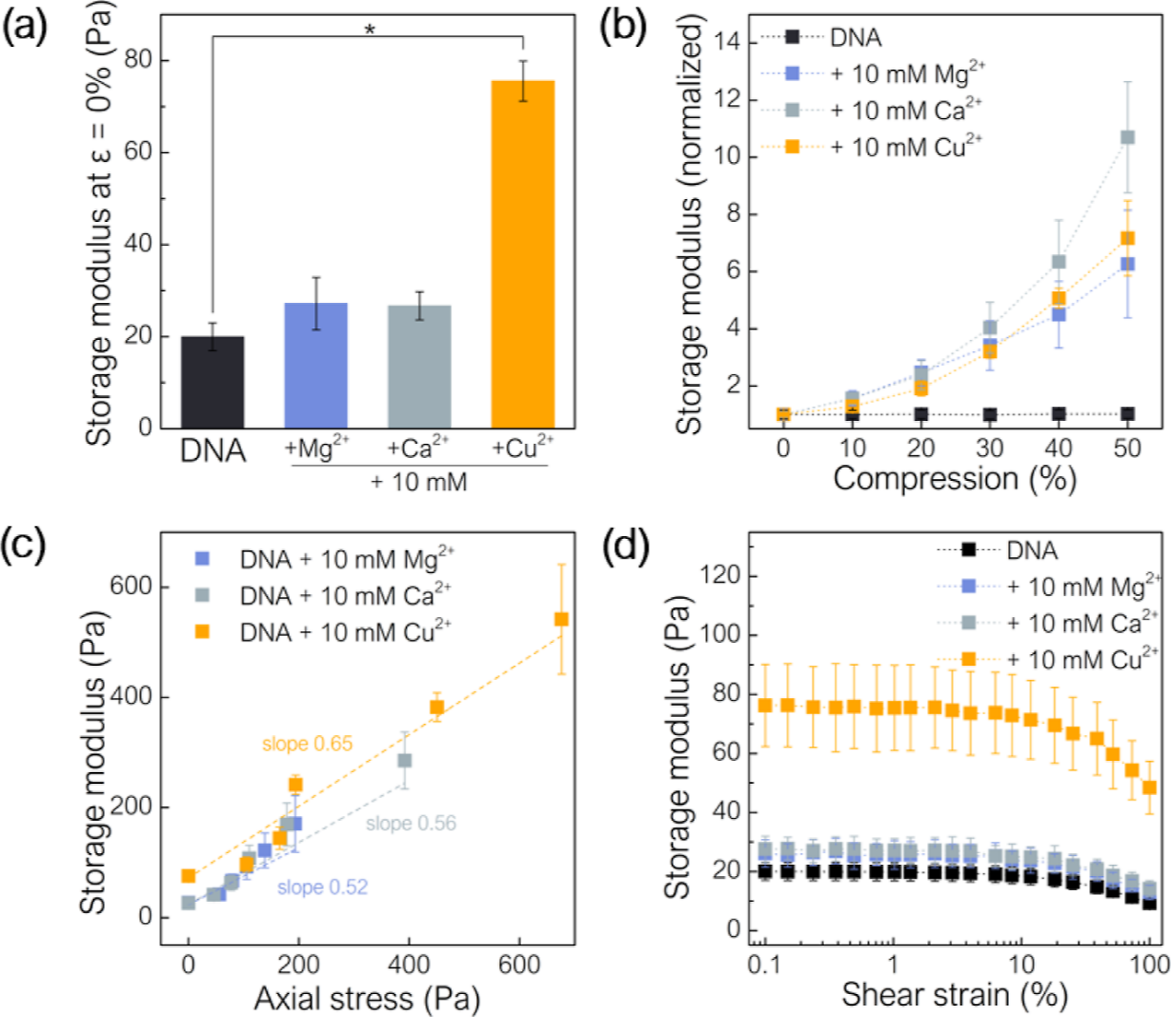
Rheological properties of DNA solutions with 10 mM bivalent Mg^2+^, Ca^2+^, and Cu^2+^ ions, (a) uncompressed sample storage modulus determined during oscillatory shear with constant amplitude γ = 1% and constant frequency *f* = 1 Hz, (b) normalized storage modulus at different compression levels, (c) storage modulus as a function of axial stress, and (d) storage modulus as a function of shear strain amplitude. Compression-stiffening was observed in the DNA network obtained by the interaction of bivalent ions. (*) indicates statistical significance (*p* < 0.05).

Biofilms are complex structures, and the observed rheological effects are the result of multiple overlapping mechanisms. As shown here, the compression-stiffening phenomenon can be recreated by the interaction of cellular components with matrix components such as DNA. We consider other possible contributors such as cross-linking of EPS components. Double-stranded DNA is a highly negatively charged polymer, and therefore cross-linking in DNA solutions can occur with the appropriate concentration of counterions. Rheological data after the addition of 10 mM of the bivalent cations magnesium (Mg^2+^), calcium (Ca^2+^), or copper (Cu^2+^) show that cross-linking likely occurs, as evidenced by an increase in storage modulus during compression (Figure 5). The axial stress and storage modulus relaxation over time is shown in Figure S4c,d.

The storage modulus of uncompressed DNA samples in monovalent salt (Figure 5a) is approximately 20 Pa and increases to a greater extent in the presence of Cu^2+^ ions, demonstrating a higher degree of cross-linking. Additionally, the presence of Mg^2+^, Ca^2+^, and Cu^2+^ cations changes the mechanics of DNA solutions during compression, increasing the degree of compression-stiffening (Figure 5b). In the presence of Mg^2+^ ions, the stiffness of DNA networks at 50% compression increases more than sixfold over 0% compression, for Ca^2+^, almost 11 times, and for Cu^2+^ increases more than 7 times. As for DNA with 50% cell volume, for DNA with 10 mM bivalent ions, the storage modulus as a function of axial stress is linear with a slope of 0.52 for Mg^2+^, 0.55 for Ca^2+^, and 0.65 for Cu^2+^ (Figure 5c). The presence of the bivalent ions maintains the shear-softening effect, with the low shear storage modulus being correspondingly higher and the percentage reduction in stiffness being less (<50%) than for the DNA and cell compositions (Figure 5d). In addition, Mg^2+^ or Ca^2+^ ions in the same concentration do not affect the stiffness of the PA biofilm, but Cu^2+^ ions significantly increase the storage modulus (Figure S5).

**Figure 6:**
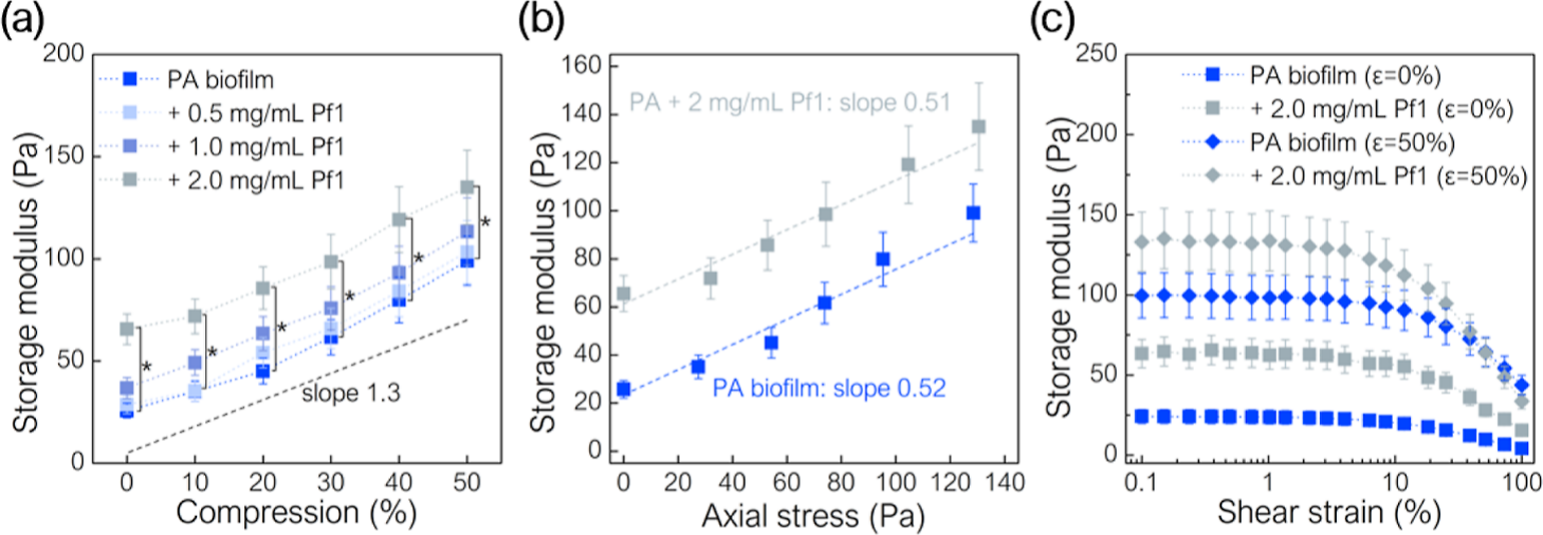
PA biofilm storage modulus with and without Pf1 bacteriophage (a) as a function of compression (the slope line shown is the reference mean line), (b) as a function of axial stress, and (c) as a function shear strain. PA biofilm stiffens in the presence of Pf1. (*) indicates statistical significance (*p* < 0.05).

To understand the basis of biofilm defense mechanisms, such as compression-stiffening, we focused our attention on biofilms made by *P. aeruginosa* Xen 5, as this species is responsible for persistent lung infections in adult CF patients. PA forms dense, difficult to remove, pathogenic communities that colonize airway surfaces so that most antibiotics have limited penetration and must be used at higher concentrations to be effective in comparison to planktonic PA cells. One reason for this may be the complex, mutualistic relationship of temperate bacteriophages,^33^ such as Pf1, with PA bacterial cells that cause an increase in biofilm density and virulence. James et al.^34^ indicate that temperate phages of *P. aeruginosa* retain lytic activity after prolonged periods of chronic infection in the CF. Pf1 virions have a diameter of 6–7 nm and a length of up to 2 μm,^35^ they are negatively charged, and their mechanical properties are similar to those of the polymer fibers of the cytoskeleton.^36^ Without a biofilm structure, they can form liquid crystal structures.^37^ Because they can interact mechanically with biofilm components, we examined how Pf1 can affect the viscoelastic properties of PA biofilms during compression. Storage moduli as a function of compression for the PA biofilm with and without the addition of Pf1 bacteriophages were determined (Figure 6a). A Pf1 concentration of 2.0 mg/mL significantly increases the stiffness of the PA biofilm (up to 255% without compression and up to 136% with compression) but does not change the linearity of the relationship. Moreover, the PA biofilm storage modulus without and with Pf1 is linearly related to the axial stress during compression with a slope of ∼0.5 (Figure 6b). The addition of Pf1 increased biofilm stiffness but does not affect shear-softening, both without and with compression (Figure 6c).

## Discussion

Our results demonstrate that the mechanical phenomena of compression-stiffening and shear-softening observed in biofilms can be recapitulated and modulated by changing the composition of DNA and microbial cells. During rheological testing of naked DNA, the storage modulus remained constant with increasing compression but in DNA embedded with a 30% or greater number of cells the storage modulus increases due to compression, and this effect is more pronounced for *P. aeruginosa* Xen 5 than for *S. aureus* Xen 30 or *C. albicans* 1408. The presence of cells changes the mechanics of DNA, presumably by providing additional cross-links and limiting the space into which DNA can move during macroscopic deformation. These data suggest that, like eDNA in the biofilm,^4^ DNA can adsorb to the cell surface causing partial cross-linking. Another factor may be the different shape of the cells which results in a different redistribution of mechanical stress during compression. An important observation is the linear relationship of storage modulus and the axial stress generated during compression: *G*′(σ) = *m*σ + μ, with *m* ∼ 0.6 – 0.8 (depending on the type of cells in the DNA network). Engstrom et al.^22^ devoted their attention to this issue, proposing two approaches to the interpretation, based on the Barron and Klein^38^ or Birch’s^39^ theory.

**Figure 7:**
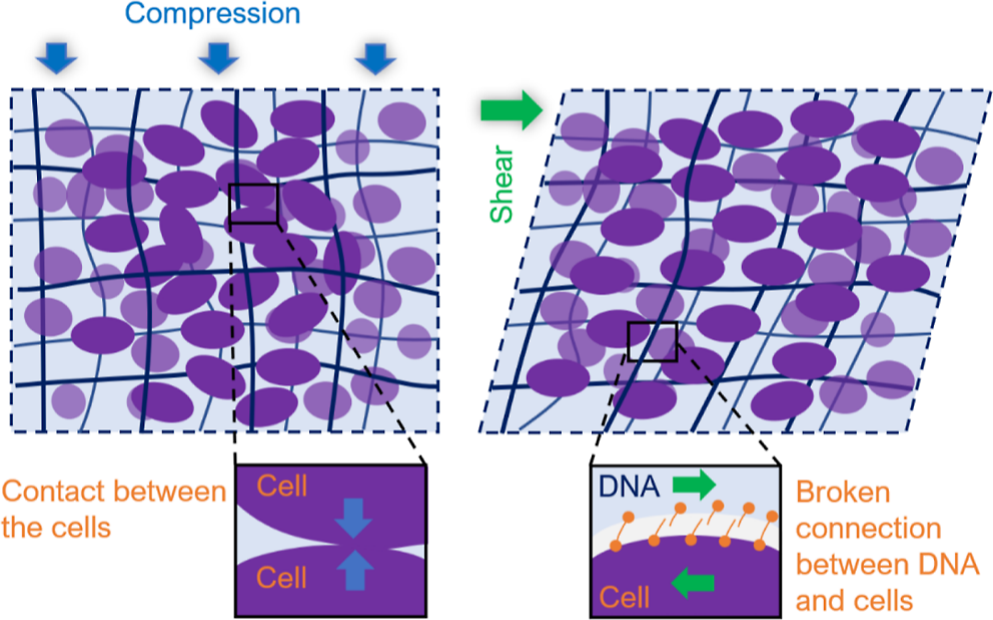
Schematic explanatory model of compression-stiffening (left) and shear-softening (right) for DNA and cell compositions. During compression, the reorganization of incompressible cells in the material takes place, which forces contact between the cells and a significant increase in mechanical resistance. During shearing, DNA strands and cells are shear oriented, and the connections between DNA and cells are broken.

Although compression-stiffening of biofilm is not well-studied, it has been well investigated for tissues.^10,22,40,41^ By adopting the biofilm as a tissue archetype,^42^ and more precisely, a material composed of relatively stiff particle inclusions in a polymer matrix, we can apply tissue mechanical models to the biofilm to explain the observed compression-stiffening. Most phenomenological models assume that interactions between cellular elements and the matrix of extracellular substances are essential. Shivers et al.^21^ suggested a mechanism where during compression, the inhomogeneous inclusion rearrangement can induce tension in the network, causing a macroscopic transition to a tension stiffening regime. However, this mechanism can only occur with inherent shear strain stiffening, which we have not observed in the semiflexible composition of DNA and cells. Perepelyuk et al.^40^ described a model, developed for the liver tissue, which involved the incompressible cellular elements and the porous, compressible phase of the extracellular substances. During compression, the fluid flows out from the matrix, forcing contact between the cells and generating significant mechanical resistance. During shearing, the connections between the matrix and cell are allowed to break and the mechanical resistance is reduced. However, DNA systems do not have a typically fibrous phase, but the observed compression-stiffening and shear-softening is qualitatively similar (Figure 7).

Shear-softening was previously investigated for entangled DNA solutions in the large-amplitude oscillatory strain regime.^43^ The decomposition of the stress signal into harmonics showed that at large strains, DNA solutions showed non-zero and increasing higher harmonics, indicating an intracycle strain-stiffening, despite the observed decrease in the storage modulus (related to the first harmonic). Similar observations for large deformations in biofilms were described by Jana et al.^44^ Shear softening can occur both due to the orientation of macromolecules and cells toward shear and breaking the cell attachments to DNA chains.

Our data show that biofilm mechanics may involve mechanisms beyond the interaction of cellular elements with the polymer matrix, as is evident by the mechanical effects of cross-linking in the presence of bivalent ions and the interaction of biofilm components with filamentous Pf1 bacteriophages. The contribution of the latter should be studied in more detail to explain possible structural changes taking place in the biofilm, such as the formation of liquid crystals.^37^ Our current study shows that Pf1 significantly stiffens the biofilm, suggesting that antimicrobial therapies should target Pf1. James et al.^34^ suggest that therapies that induce the lytic cycle of the temperate phage may be a beneficial alternative or addition to standard antibiotic treatment. The observed compression-stiffening in DNA solutions containing a high concentration of cations is similar to the behavior previously described in agarose gels,^22^ among others. Bivalent cations, like calcium and magnesium, are well-established gelling agents for EPS biopolymers, such as alginates used in bio-printing.^45,46^ Here, we show that magnesium and calcium aggregate DNA in a similar way, with calcium having a slightly greater impact on the degree of compression-stiffening. Copper ions, on the other hand, increase the stiffness of DNA gels and may be an effective gelling agent for DNA-based hydrogels. Extensive research on the effect of multivalent cations on the viscoelastic properties of filamentous anionic biopolymers (which includes DNA) is presented by Cruz et al.,^47^ who show that various bivalent cations aggregate polyelectrolytes, transition-metal ions are more effective than alkaline earth metal ions, and their efficiency increases with increasing atomic mass.

## Conclusions

We conducted shear rheometry studies at various levels of compression for *P. aeruginosa* Xen 5, *S. aureus* Xen 30, and *C. albicans* 1408 biofilms as well as DNA networks embedded with cells of these microorganisms. The results show compression-stiffening and shear-softening, which are also observed in other biological materials such as tissues. Compression-stiffening was not observed for DNA solutions containing little to no cells, but the addition of 30% cells or greater recapitulates the behavior, likely due to the interaction of cells with matrix fibers. Interestingly, the extent of compression-stiffening is cell type-dependent, but it is difficult to assess what factor contributes to such rheological behavior and requires further study. Biofilm mechanics is largely DNA-dependent, which implies that interference with DNA may affect biofilm integrity and prevent infection. This shows that standard DNase therapies can not only be used for CF lung infection but also might be considered in other biofilm-related infections. The DNA structure and the presence of filamentous components such as bacteriophages also affect the mechanics of biofilms. Compression-stiffening can be simulated in solutions by cross-linking using a high concentration of bivalent cations. The higher the atomic weight of the cation, the greater the cross-linking. Our results indicate that the stiffness of the biofilms of *P. aeruginosa* Xen 5 increases with the concentration of bacteriophages. The present work expands the current knowledge of biofilm mechanics and biopolymer rheology and may have important implications in the development of new biomimetic materials.

## Supporting Information Available

Storage and loss modulus as a function of frequency; absolute values of PA, SA, and CA biofilm storage modulus as a function of compression and shear strain; morphology of cells used in the test; axial stress and storage modulus relaxation over time at different compression levels; and normalized storage modulus of uncompressed and compressed PA biofilm with bivalent ions (PDF)
