# Supplementary material for: Modulation of Biofilm Mechanics by DNA Structure and Cell Type
Source: ACS Biomater Sci Eng. 2022 Oct 27;8(11):4921–9. doi: 10.1021/acsbiomaterials.2c00777 (PMC9667457; doi:10.1021/acsbiomaterials.2c00777)
Supplement: Supplementary file 1 — ab2c00777_si_001.pdf [file ab2c00777_si_001.pdf]

Supporting Information for

Modulation of biofilm mechanics by DNA structure and cell type

Dawid Łysik[1], Piotr Deptuła[2], Sylwia Chmielewska[2], Karol Skłodowski[2], Katarzyna Pogoda[3], LiKang Chin[4], Dawei Song[5], Joanna Mystkowska[1], Paul A. Janmey[5], Robert Bucki[2]

[1]Institute of Biomedical Engineering, Bialystok University of Technology, Bialystok, Poland
[2]Department of Microbiological and Nanobiomedical Engineering, Medical University of Bialystok, Poland
[3]Institute of Nuclear Physics, Polish Academy of Sciences, Krakow, Poland
[4]Department of Biomedical Engineering, Widener University, Chester, PA, USA
[5]Institute for Medicine and Engineering, University of Pennsylvania, PA, USA

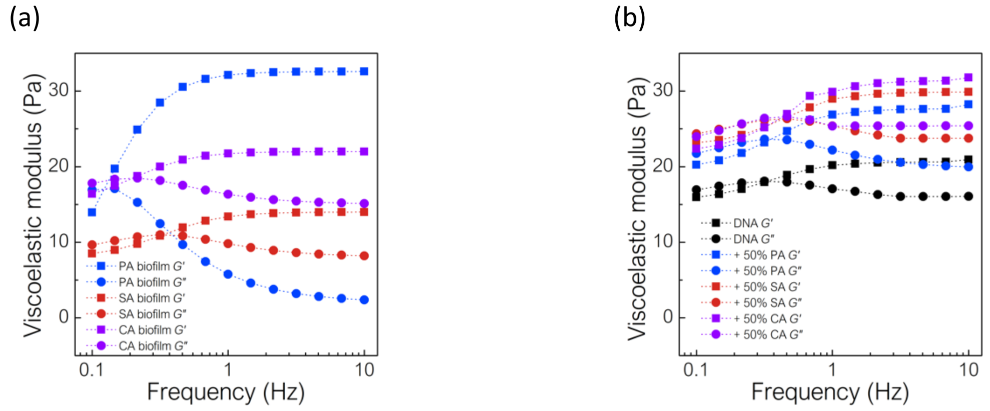

Figure S1. Storage (*G*′) and loss (*G*″) modulus as a function of frequency (**a**) of PA, SA, and CA biofilm; (**b**) DNA-cell systems.

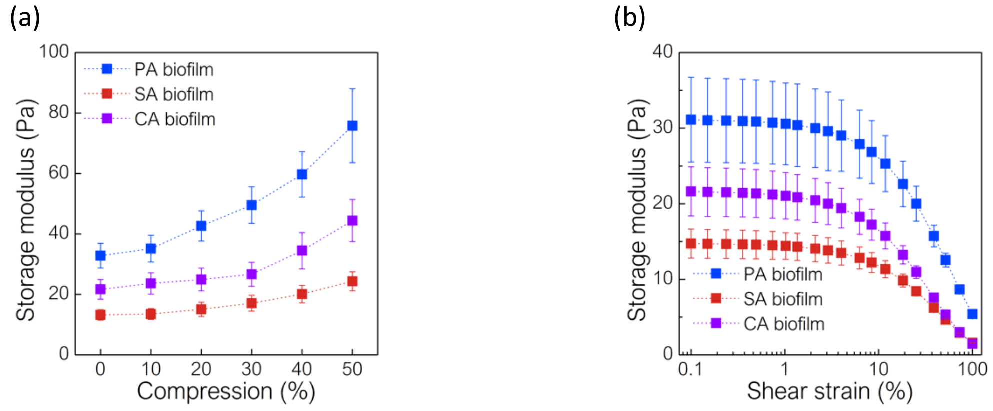

Figure S2. Absolute values of PA, SA, and CA biofilm storage modulus as a function of (**a**) compression; (**b**) shear strain.

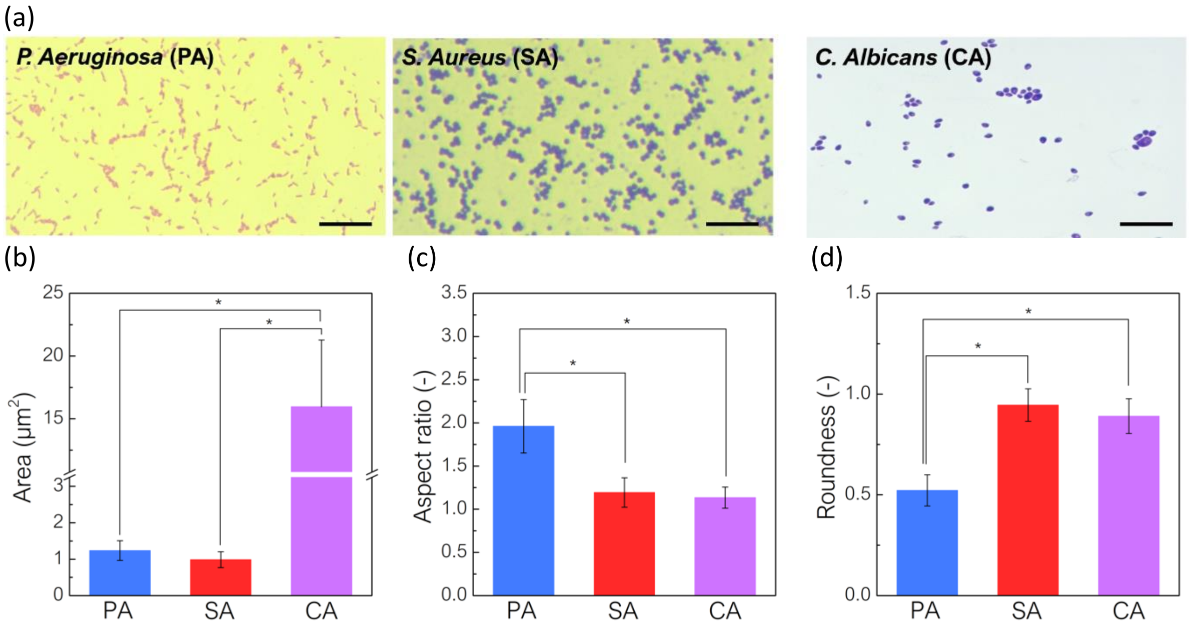

Figure S3. Morphology of cells used in the test: (**a**) microscopic photos of the cells; (**b**) mean area of a single cell; (**c**) their aspect ratio (long to short axis); and (**d**) roundness (the ratio between the circles inscribed and described on the cells). Measurements were carried out on 100 selected cells of a given group. (*) indicates statistical significance (p<0.05).

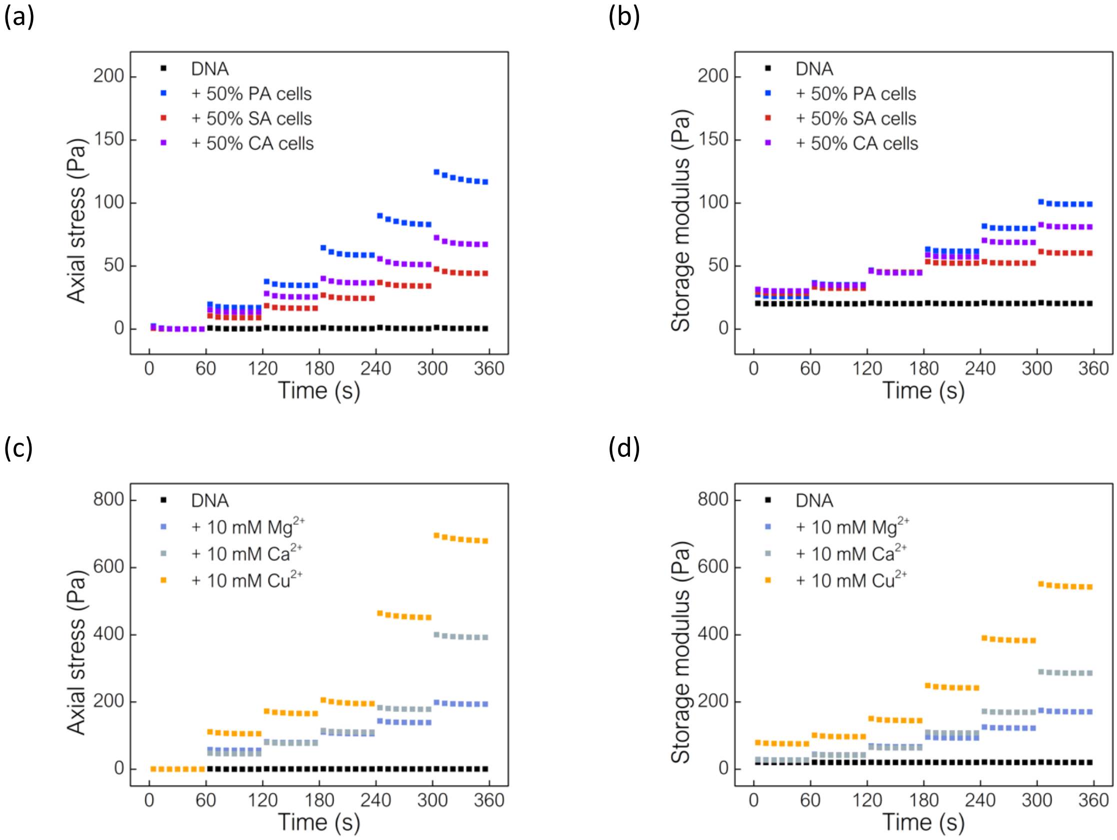

Figure S4. Axial stress and storage modulus relaxation over time at different compression levels for (**a, b**) DNA-cells, (**c, d**) DNA crosslinked by bivalent ions.

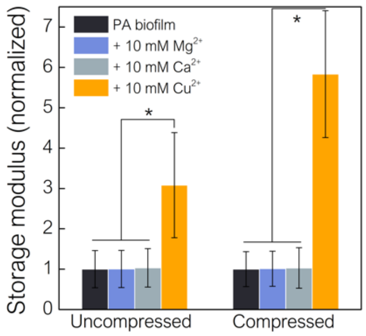

Figure S5. Normalized storage modulus of uncompressed and compressed PA biofilm with bivalent ions. (*) indicates statistical significance (p<0.05).
